# Interleukin-2 Receptor and Angiotensin-Converting Enzyme as Markers for Ocular Sarcoidosis

**DOI:** 10.1371/journal.pone.0147258

**Published:** 2016-01-22

**Authors:** Enken Gundlach, Michael Marcus Hoffmann, Antje Prasse, Sonja Heinzelmann, Thomas Ness

**Affiliations:** 1 University Eye Hospital Charite, Berlin, Germany; 2 Eye Center, University Hospital Freiburg, Freiburg, Germany; 3 Institute for Clinical Chemistry and Laboratory Medicine, University Hospital Freiburg, Freiburg, Germany; 4 Department of Pneumology, University Hospital Freiburg, Freiburg, Germany; 5 Department Respiratory Medicine Medical School Hannover, Hannover, Germany; 6 Clinical Research Center Fraunhofer ITEM, Hannover, Germany; Oregon Health & Science University, UNITED STATES

## Abstract

**Purpose:**

To study the impact of soluble IL2 receptor (sIL2R), chest x-ray (CxR), and angiotensin-converting enzyme (ACE) as markers for sarcoidosis in uveitis patients.

**Design:**

Retrospective study.

**Methods:**

Serum concentrations of sIL2R and ACE were measured in patients with active uveitis. Those with elevated sIL2R and /or ACE values were examined for suspected systemic sarcoidosis.

**Main Outcome Measure:**

Our main outcome parameters were the specificity and sensitivity of sIL2R, CxR and ACE in screening for ocular sarcoidosis.

**Results:**

We measured 261 patients with uveitis for sarcoidosis using sIL2R and ACE between January 2008 and November 2011; sarcoidosis was been diagnosed using other tests (e.g. computer tomography, brochoalveolar lavage, biopsy) in 41 of 53 patients with elevated sIL2R values (>639 U/ml) and in one patient with normal sIL2R (582 U/ml). Their mean sIL2R value was 1310 U/ml, extending from 582 to 8659 U/ml. Only 9 patients, however, presented elevated ACE (>82 U/l). Their mean ACE value was 116.4 U/l, ranging from 84.1 to 175.5 U/l. IL2R specificity was 94% with 98% sensitivity. In contrast, ACE had a specificity of 99.5%, but a sensitivity of only 22%; the chest x-ray had a specificity of 100% with 50% sensitivity in detecting sarcoidosis. We observed the entire spectrum of uveitis: sixteen patients suffered from anterior, 8 from intermediate, 16 from posterior, and 2 from panuveitis.

**Conclusions:**

An elevated level of soluble IL2R suggests sarcoidosis with uveitis more convincingly than ACE, making sIL2R a more effective marker parameter for sarcoidosis than ACE or chest x-ray in uveitis patients.

## Introduction

Sarcoidosis or Morbus Boeck is a multisystem disorder of unknown cause[1,2,3]. The organs involved are histologically characterized by non-caseating granulomas. The organs most commonly affected are the mediastinal and hilar lymph nodes and the lungs (50–90%)[4,5,6]. Ophthalmic involvement occurs in 30–60% of patients with sarcoidosis [7,8,9]. In some cases sarcoidosis predominantly affects the eye. According to the International Workshop of Ocular Sarcoidosis (IWOS) the term “ocular sarcoidosis” is used to describe such cases[7,10].

Sarcoidosis is distributed worldwide and usually starts between the ages of 20 and 40 years. Its prevalence in the USA is three times higher in the Afro-American population than in the Caucasian population[11,12,13,14,15,16].

Untreated sarcoidosis may be fatal, and intraocular involvement can lead to blindness[9]. The ophthalmologist’s role is important, since uveitis may be the first manifestation of sarcoidosis[16,17,18,19].

However, diagnosing it can be challenging[20]. The gold standard is histopathological proof from biopsy tissue[21,22]. As intraocular tissues are rarely biopsied due to the risk of vision loss, biopsies from nonocular tissues such as lung, skin and peripherial lymph nodes have been taken into account[2,23]. No other specific clinical symptom or investigation is diagnostic. For this reason, and depending on the extent of work-up, sarcoidosis is probably under-diagnosed in uveitis patients.

In 2009, an international group of uveitis specialists therefore defined diagnostic criteria for ocular sarcoidosis, choosing criteria enabling the ophthalmologist to make the diagnosis of ocular sarcoidosis without the invasive diagnostic procedures needed for histological proof. These criteria included signs of intraocular inflammation and laboratory investigations[7].

Not mentioned in these criteria is the soluble interleukin-2 receptor (sIL2R), a serum marker widely used for the diagnosis of and as an activity marker in pulmonary sarcoidosis[24,25,26,27,28,29,30].

Although its exact pathogenesis is unknown, sarcoidosis is characterized by T-cell activation[31,32,33]. Those activated T-cells express the interleukin-2 receptor on their surface (CD25, 55-kDa/75-kDaheterodimer) and release a soluble form of the 55-kDa chain called soluble IL2 receptor (sIL2R)[34]. The specific role of sIL2R in the immune response is not yet completely understood, but an elevated serum sIL2R level is known to correlate with the activity of T-cell-mediated diseases[35,36]. Such T-cell mediated diseases are systemic lupus erythematosus, juvenile idiopathic arthritis or sarcoidosis[37,38]. The impact of sIL2R as a marker of disease activity in patients with systemic or pulmonary sarcoidosis has been shown in previous studies[39,40]. Soluble IL2-receptor is more sensitive than the serum marker ACE in detecting pulmonary sarcoidosis[41]. Further studies demonstrated that sIL2R might be useful both as a marker of severity and as a prognostic factor in systemic or pulmonary sarcoidosis[24,25].

The aim of this study was to evaluate the potential clinical usefulness of sIL2R as a screening parameter for sarcoidosis with uveitis in comparison to established markers (chest x-ray, ACE).

## Methods

### Patients

Between January 2008 and November 2011, 600 patients were seen with uveitis for the first time in the uveitis section at the Eye Center, University of Freiburg. Out of these 261 were tested for sIL2R and ACE. In 53 of those we detected elevated sIL2R. All patients suspected of having intraocular sarcoidosis were referred to a respiratory specialist (pulmonologist).

Medical data were reviewed retrospectively. We analyzed age, gender, comorbidity, current medications (including ACE inhibitors) and visual acuity.

Anatomical location of the uveitis and its severity were classified according to the standardisation in uveitis nomenclature (SUN) criteria[42]. In the absence of a real “gold standard”, namely tissue biopsy from intraocular tissue, intraocular sarcoidosis was diagnosed according to recommended criteria from the International Workshop of Ocular Sarcoidosis (IWOS)[7].

We also collected chest radiographs, computer tomographies, bronchoalveolar lavage results, biopsies, and laboratory data. In accordance with the European Respiratory Society patients with alveolar lymphocytes ≥ 20% were considered to present a pathology[43]. Due to the study’s being retrospective, these specific tests were not available for all patients. An examination by an internal medicine specialist was also considered.

Institutional Review Board (IRB)/Ethics Committee approval was obtained (Ethik-Kommission der Albert-Ludwigs-Universität Freiburg (No.69/14)). According to the IRB requirements patient records and information were anonymized and de-identified prior to analysis.

### Analysis of Serum Parameters

ACE levels were determined by spectrophotometric assay using the synthetic tripeptide substrate N-[3-(2-furyl)acryloyl]-L-phenylalanylglycylglycine (FAPGG), which is catalyzed by ACE to FAP and glycylglycine (Roche). This hydrolysis results in a decrease in absorbance at 340 nm.

Soluble IL2R was measured via a solid-phase two-site chemiluminescence immunometric assay (Siemens, Germany). Soluble IL2R in the sample binds to the solid-phase antibody and reagent antibody (coupled with alkaline phosphatase), forming a sandwich complex. A chemiluminescent signal is generated in proportion to the bound enzyme. According to the manufacturer, the threshold value for ACE is 82 U/mL and 639 U/ml for sIL2R, respectively.

### Statistics

Contingency table to achieve specificity, sensitivity, positive predictive value and negative predictive value of ACE, chest x-ray and sIL2R were calculated using Prism from the Graph Pad. The Youden Index, a marker of the performance of a diagnostic test, was also provided[44]. Furthermore, we compared sIL2R values between controls and sarcoidosis patients using the Wilcoxon-Mann-Whitney-test.

## Results

### Patient characteristics

The patients with elevated sIL2R were classified in two categories: the first included all patients with ocular sarcoidosis, including 41 patients with elevated sIL2R and one patient with normal sIL2R value (582 U/ml) and in the second, those with elevated sIL2R and no evidence of sarcoidosis.

**Fig 1:**
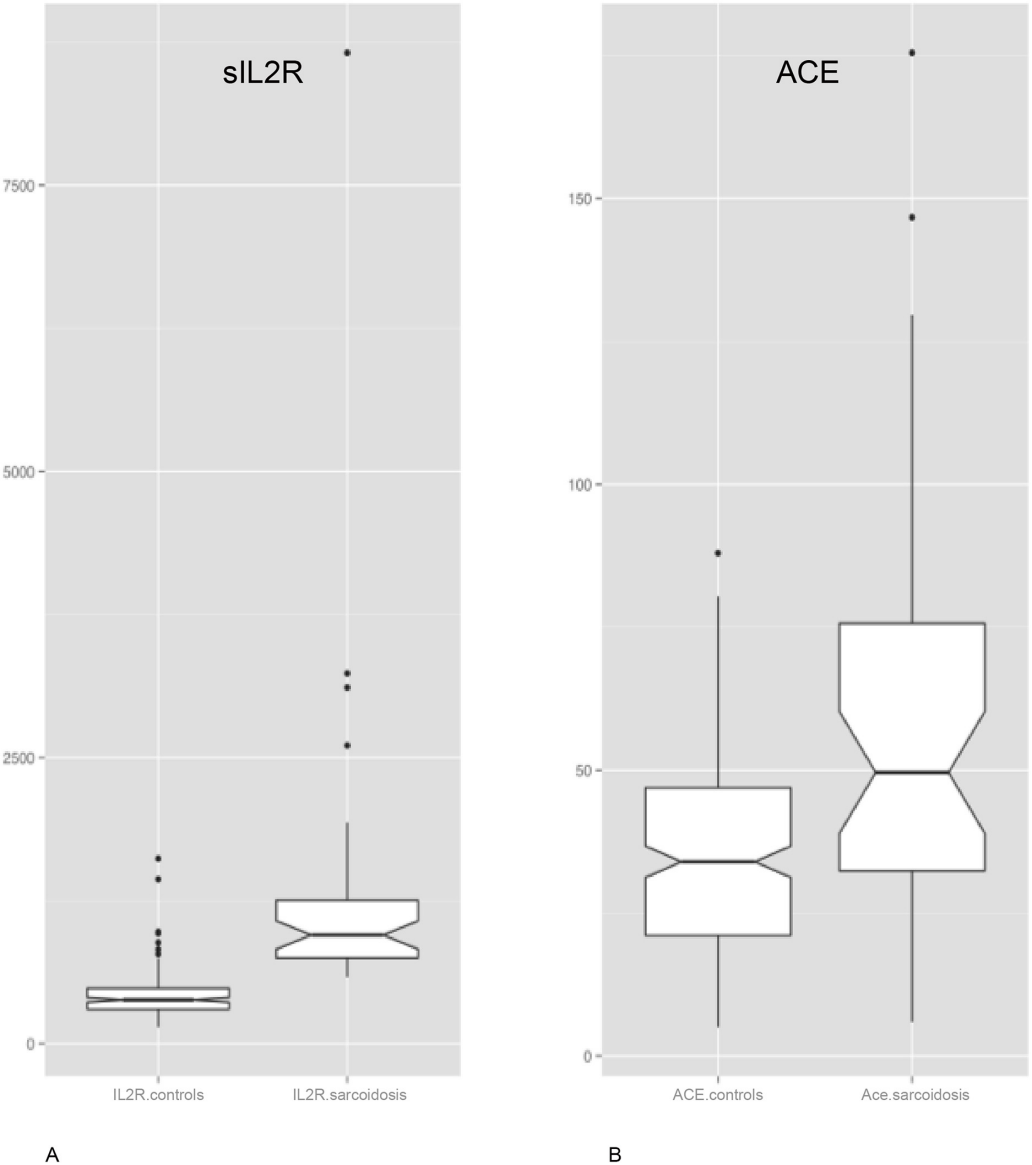
Soluble IL2R levels (a) (p < 0.0001) and ACE levels (b) (p < 0.001) for controls (all uveitis patients/no sarcoidosis) and sarcoidosis patients with uveitis (S1 Dataset).

The male/female ratio did not differ between these categories, and the median age was similar (Table 1). Due to the study being retrospective, sarcoidosis had been diagnosed in a variety of ways; the diagnosis of pulmonary sarcoidosis was confirmed by chest x-ray in 13 patients, by CT scan in 16 patients, by bronchoalveolar lavage in 18 patients[45], and by tissue biopsy in 10 patients, respectively. Mean sIL2R value was 1310 U/ml (SEM ± 203; SD ± 1298) in the uveitis patients with sarcoidosis, while only 918 U/ml (SEM ± 89) in those uveitis patients without sarcoidosis but with elevated sIL2R (second group) (Table 1). Compared with all the uveitis patients (mean sIL2R = 414 U/ml; SEM ± 13, SD ± 180), the mean sIL2R value in sarcoidosis patients was significantly elevated (1310 U/ml) (p<0.0001) (Fig 1a).

**Table 1:** Characteristics of patients with elevated sIL2R: gender, angiotensin converting enzyme (ACE), chest x-ray, high resolution CT scan, bronchoalveolar lavage (BAL) (pos BAL = ≥ 20% alveolar lymphocytes[43]), tissue biopsy, Standard error of the mean (SEM).

	Sarcoidosis* with uveitis	Uveitis without sarcoidosis*
	n = 42	n = 12
gender (% female)	64%	75%
age years (median)	21–86 (56)	15–79 (57))
patients with elevated angiotensin-converting enzyme (>82 U/l) (n)	9	0
chest x-Ray (abnormal) (n)	13	0
pos. CT-Scan (n)	16	0
pos BAL (n)	18	0
pos. tissue biopsy (n)	10	0
sIL2R (U/ml)		
mean	1310	918
range	582–8659	642–1618
SEM	± 203	± 89
ACE (U/ml)		
mean	59	35
range	6–176	5–81
SEM	± 6.2	± 7.3

* according to the International Workshop of Ocular Sarcoidosis criteria (IWOS)

Mean ACE value was 59 U/ml in the sarcoidosis patients with uveitis (n = 41) while that of the uveitis patients without sarcoidosis (n = 220) was just 35 U/ml (Fig 1b) (p<0.001). There are two patients in the ocular sarcoidosis group, and one patient in the no sarcoidosis group taking an ACE inhibitor. Since in real life some uveitis patients will be taking ACE inhibitors during screening, we did not eliminate them from the calculation. However, had we considered those patients in our analysis, it would have made no difference in our results.

The patients with elevated sIL2R/no sarcoidosis suffered from idiopathic non-granulomatous uveitis (6), from systemic autoimmune disease (3) (undifferentiated systemic autoimmune disease (2), lupus erythematosus (1)), mycoplasma infection (1), HLA B27 associated uveitis (1) or TINU syndrome (tubulointerstitial nephritis and uveitis) (1).

### Sarcoidosis diagnosis

Based on results from the chest x-rays, CT scans, tissue biopsies and IWOS criteria, we detected 42 sarcoidosis patients with uveitis. A diagnosis of definite ocular sarcoidosis was made in 10 patients, presumed ocular sarcoidosis in 12 patients, and probable ocular sarcoidosis in 19 patients. One patient was diagnosed with possible ocular sarcoidosis (Table 2).

**Table 2:** Number of patients according to the International Workshop of Ocular Sarcoidosis criteria (IWOS).

definite intraocular sarcoidosis	10
presumed intraocular sarcoidosis	12
probable intraocular sarcoidosis	19
possible intraocular sarcoidosis	1

We classified these patients according to the SUN criteria. Sixteen suffered from anterior uveitis, 8 from intermediate uveitis, 16 from posterior uveitis and two from panuveitis (Table 3).

**Table 3:** SUN classification of the sarcoidosis patients with uveitis (Number of patients).

	sarcoidosis patients
anterior uveitis	16
intermediate uveitis	8
posterior uveitis	16
panuveitis	2

### Sensitivity, specificity, positive predictive value, negative predictive value, Youden Index

Specificity was high for the chest x-ray, sIL2R and ACE. It was lowest for sIL2R at 94%. In contrast, the sIL2R sensitivity was 98%, chest x-ray 50%, and ACE only 22%. Thus positive and negative predictive values were favorable for all factors, but Youden Index was only favorable for sIL2R (0,92) (Table 4, S1 Table).

**Table 4:** Contingency tables: evaluation of sensitivity, specificity, positive predictive value (ppv), negative predictive value (npv) and Youden index[44] in screening for sarcoidosis with uveitis.

	sensitivity	specificity	Youden Index	positive predictive value	negative predictive value
sIL2 R*	98%	94%	0.92	0.77	0.99
chest x-ray*	50%	100%	0.50	1.0	0.91
ACE*	22%	99.5%	0.22	0.9	0.87

* p<0.0001; soluble Interleukin 2 Receptor (sIL2R); Angiotensin converting enzyme (ACE)

## Discussion

The eye is a potential primary and/or presenting site for the manifestation of sarcoidosis. Ophthalmologists play a critical role in establishing its diagnosis[19,46]. In 2009, an international group of uveitis specialists assessed diagnostic criteria for ocular sarcoidosis. In addition to the clinical presentation and radiological signs, they also assessed laboratory results. As predominant ocular sarcoidosis is rare, its clinical manifestation easily overlooked, and as current radiological findings and biopsy are invasive and often unavailable, a sensitive laboratory marker to screen for ocular sarcoidosis would be a substantial bonus. Members of the international Workshop on Ocular Sarcoidosis recommended elevated serum angiotensin converting enzyme (ACE), elevated serum lysozyme and abnormal liver enzyme tests. But none of those tests is highly sensitive[7,47].

Serum angiotensin converting enzyme (ACE) is probably the most common laboratory test for sarcoidosis, revealing an approximate sensitivity of 57–73% and a specificity of 90% in the literature, but only 22% sensitivity and 90% specificity in our patients[48,49]. Even in biopsy-proven sarcoid uveitis cases, only 61.7% presented increased ACE values[50].

ACE is secreted by epithelioid cells and macrophages and is thus a marker for the general burden of granulomas[51]. ACE is indeed higher in clinically-active disease than in inactive disease, and it does correlate with the disease´s extent, but there is no correlation between initial ACE levels and the response to therapy or prognosis[52]. In addition, the ACE value should be interpreted with respect to the genotype[53].

An ideal marker would be one that is easy to obtain at any time and safe for the patient. The serum sIL2R value fulfills these criteria and is already frequently used in the clinical routine for diagnosing pulmonary and systemic sarcoidosis[26,29,30]. It is described as a marker of disease activity[25,39,54]. Soluble IL2R is also known to be a marker for extrapulmonary involvement[54,55], its value as a disease marker in ocular sarcoidosis was first reported by our group in 2012, and confirmed by others later [56,57].

In the present retrospective study, we assessed serum sIL2R in a group of patients with ocular sarcoidosis. On the basis of our data, we can claim that soluble IL-2R is a useful screening marker for sarcoidosis with uveitis. All kinds of intraocular uveitis (anterior, intermediate, posterior, panuveitis) were detected as being associated with subclinical pulmonary sarcoidosis. Furthermore, we measured a sensitivity of 22% and a specifity of 90% for ACE. In direct comparison, IL2R seems to be the more appropriate screening tool for the diagnosis (sensitivity 98% // specificity 94%// Youden Index 92%). Soluble IL2R is a parameter that indicates the extent of T-cell activation and has been known since the early eighties[35,36]. Elevated levels have therefore been measured in a variety of diseases such as systemic lupus erythematosus and juvenile idiopathic arthritis[37,38]. Half of our 12 patients without sarcoidosis but elevated sIL2R values suffered from such diseases (e.g. systemic autoimmune diseases).

An increased IL2R level has been reported to be associated with uveitis. Few studies have demonstrated a relationship between sIL2R levels in peripheral blood and in aqueous humor with uveitis[58,59,60,61]. Martin et al measured a mean sIL2R in the peripheral blood of 434 U/ml. This value corresponds approximately to our mean in all the uveitis patients we tested (414 U/ml)[60]. Arocker-Mettinger et al also detected a slightly elevated sIL2R value in their patients[58]. There were, however, no sarcoidosis patients involved in either study, unfortunately. Torun et al also noted elevated sIL2R values in their series of uveitis patients, but did not monitor them for sarcoidosis[62]. Papadaki et al recently negated any correlation between sIL2R and an associated autoimmune systemic disease[63], but they did report high sIL2R values in patients with sarcoidosis also. Murray and Young found the highest levels in patients with sarcoidosis[61].

There are some limitations in our study; it is only a retrospective, single-center study. As mentioned above, there is no real gold standard available, thus we applied the IWOS criteria. Taking this into account, you will never even in a prospective study detect all true positives and false positives. Therefore, there will be always some degree of uncertainity.

In addition, the performance of a test depends tremendously on the population in which it is assessed. The specificity would be less in a population with lower incidence of sarcoidosis.

Nevertheless, our results clearly indicate that sIL2R levels are superior to ACE levels and CxR for identifying patients with sarcoidosis even if the study has the aforementioned limitations.

In summary: based on our data; we recommend adding the measurement of sIL2R in the routine diagnostic work-up of patients with uveitis as an additional screening parameter for intraocular sarcoidosis. Moreover, already established biomarkers such as ACE and lysozyme may be replaced by sIL2R.

## Supporting Information

S1 Dataset sIL2R/ACE values. (XLSX)

S1 Table contingency tables: evaluation of sensitivity, specificity, positive predictive value (ppv), and negative predictive value (npv) in screening for ocular sarcoidosis. * p<0.0001; soluble Interleukin 2 Receptor (sIL2R); Angiotensin converting enzyme (ACE).
(DOC)
