# Supplementary material for: Interleukin-2 Receptor and Angiotensin-Converting Enzyme as Markers for Ocular Sarcoidosis
Source: PLoS One. 2016 Jan 22;11(1):e0147258. doi: 10.1371/journal.pone.0147258 (PMC4723126; doi:10.1371/journal.pone.0147258)
Supplement: S1 Table — * p<0.0001; soluble Interleukin 2 Receptor (sIL2R); Angiotensin converting enzyme (ACE). (DOC) [file pone.0147258.s002.doc]

| Chest x-ray | Sarcoidosis | |  |  |
| --- | --- | --- | --- | --- |
|  | + | - |  |  |
| + | 13 | 0 | 13 | ppv 1.0 |
| - | 13 | 135 | 148 | npv 0.91 |
|  | 26 | 135 | 161 |  |
|  | sensitivity  50% | specificity  100% |  |  |

p<0.0001

| ACE | Sarcoidosis | |  |  |
| --- | --- | --- | --- | --- |
|  | + | - |  |  |
| + | 9 | 1 | 10 | ppv 0.9 |
| - | 32 | 219 | 251 | npv 0.87 |
|  | 41 | 220 | 261 |  |
|  | sensitivity  22% | specificity  99.5% |  |  |

p<0.0001

| sIL2R | Sarcoidosis | |  |  |
| --- | --- | --- | --- | --- |
|  | + | - |  |  |
| + | 41 | 12 | 53 | ppv 0.77 |
| - | 1 | 193 | 194 | npv 0.99 |
|  | 42 | 205 | 247 |  |
|  | sensitivity  98% | specificity  94% |  |  |

p<0.0001

S1 Table: contingency tables: evaluation of sensitivity, specificity, positive predictive value (ppv), and negative predictive value (npv) in screening for ocular sarcoidosis

* p<0.0001; soluble Interleukin 2 Receptor (sIL2R); Angiotensin converting enzyme (ACE)
